# Low-pressure headache presenting in early pregnancy with dramatic response to glucocorticoids: a case report

**DOI:** 10.1186/1752-1947-8-115

**Published:** 2014-04-03

**Authors:** Mahreen Hashmi

**Affiliations:** 1 Department of Obstetrics and Gynecology, West Virginia University, 4601 HSN, P.O. Box 9186, Morgantown, WV 26506, USA

**Keywords:** Low-pressure headache, Spontaneous intracranial hypotension, Pregnancy

## Abstract

**Introduction:**

Obstetricians are familiar with postural headaches in their postpartum patients following spinal or epidural anaesthesia. The rare occurrence in the antepartum patient without inciting event, may pose a diagnostic and treatment dilemma with resultant prolongation of disabling headaches in affected patients.

Awareness of this condition, if accurately diagnosed, may allow for earlier relief from disabling headache, which may take weeks to months to otherwise resolve.

**Case presentation:**

A case of low-pressure or postural headache (spontaneous intracranial hypotension) in a 39-year-old Caucasian patient in early pregnancy follows. She was initially misdiagnosed with migraine headache, but subsequently thought to have low-pressure headache.

**Conclusions:**

Obstetricians/neurologists need to be aware of the potential treatment options for pregnant patients. Due to the desire to limit caffeine (a standard treatment for low-pressure headache) in pregnancy, high-dose glucocorticoids may provide rapid relief without significant risk to the immunocompetent patient or the pregnancy.

Our case offers a non-interventional approach in the pregnant patient with resultant quick response to treatment without significant adverse fetal risk.

## Introduction

A relatively healthy 39-year-old Caucasian woman, in her first trimester of pregnancy, developed acute onset of postural headache without inciting event, ultimately diagnosed as low-pressure headache or spontaneous intracranial hypotension (SIH). She remained bedbound due to the severity of her symptoms, unresponsive to the usual treatment modalities - intravenous (IV) hydration and caffeine. On day 10, prednisone was initiated to which she responded quickly and completely, and which she was able to taper down and off over a month without recurrence of her headache.

Awareness of SIH is important as it often presents a diagnostic challenge, with resultant misdiagnosis and potential protracted disability. Treatments may offer limited and slow response.

We present the case of a pregnant patient who received non-interventional treatment with relatively low-risk therapy, who achieved an excellent response within hours of treatment.

## Case presentation

Our patient was a 39-year-old gravida 2 para 1 (G2P1) Caucasian woman who presented to our emergency department at 10 weeks gestation with acute onset of a throbbing occipital and right-sided headache. Additional complaints included associated nausea, vomiting, and hyperacusis. Her headache was severe, occurred within minutes of sitting or standing, but was relieved by recumbency. She denied fever or other infectious complaints and there was no recent history of trauma. This pregnancy had been otherwise uncomplicated and a first trimester ultrasound had confirmed a viable singleton intrauterine pregnancy.

An evaluation in the emergency department resulted in a presumptive diagnosis of migraine headache and she was treated with intravenous hydration and narcotics after which she was discharged home.

She returned the following day with a recurrence of her symptoms and was again treated with IV fluids and antiemetics. The results of a computed tomography (CT) scan of her head were negative. A neurology consultation was obtained and a diagnosis of SIH or low-pressure headache made on the basis that headache worsened within fifteen minutes of sitting or standing and was relieved with recumbency, with associated nausea, vomiting and hyperacusis [1].

A magnetic resonance imaging (MRI) scan was deferred due to concerns of gadolinium use in pregnancy. Complete bed rest with IV hydration and caffeine were prescribed by the neurologist for SIH. After 10 days of continuous therapy, our patient had not improved and remained unable to sit or stand without recurrence of her headache within minutes. Nausea and vomiting would ensue if she remained upright.

Prednisone was initiated at 80mg daily (for a 55kg patient) and within four hours our patient was able to sit upright without symptoms. The steroid was tapered down and discontinued over the next four weeks and our patient remained symptom-free subsequently.

## Discussion

Spontaneous intracranial hypotension is a rare condition with a prevalence of 1:50,000 persons with a female-to-male ratio of 3:1 [2]. Previous case reports in pregnancy have reported onset and recurrences noted in the first and early second trimesters [3,4].

The predominant symptoms are severe postural headache, nausea and vomiting. Other symptoms may include neck pain and stiffness, diplopia, vertigo, tinnitus, impaired hearing, convulsions, and cognitive abnormalities [5].

The accepted etiology is cerebrospinal fluid (CSF) leakage related to a dural tear where the spinal roots exit the subarachnoid space, which may occur at the thoracic or cervicothoracic junction. Meningeal enhancement on brain MRI is noted in SIH, however, the use of gadolinium-based contrast is not recommended in pregnant patients [6], though some evidence suggests this may be safe [7]. Imaging studies may not confirm a CSF leak, therefore, radiologic confirmation of the diagnosis may be inconclusive [8].

Treatment modalities that have been used in an attempt to conservatively manage this condition have included: 1) bed rest with IV hydration; 2) caffeine; and 3) epidural blood patches. Caffeine appears to increase CSF production via decreasing cerebral blood flow through adenosine receptor antagonism [1], however it is desirable to limit caffeine in pregnancy [9]. Complications due to epidural blood patch include infection, chemical inflammation, paraesthesias in the lower limbs, neck stiffness, and radicular pain [1].

Because of the rarity and unfamiliarity with this condition, SIH is often misdiagnosed as tension or migraine headaches, meningitis, and psychogenic disorder [10]. This combination of factors has led to a reported delay in diagnosis ranging from four days to 13 years with a median of five weeks and mean of 13 months [10].

Prior case citations of successful steroid use in pregnancy for SIH have included oral, lumbar epidural injection, and IV cosyntropin [11-13]. The mechanism of action of cosyntropin is unknown, but is thought to affect the flow of sodium in the choroidal membrane [11].

Given the potential complications of diagnostic and therapeutic procedures in SIH, a short course of oral steroid may obviate the need for further testing that may negatively impact the fetus.

## Conclusions

The diagnosis of low CSF pressure headache should be considered in patients who present with postural headache with or without associated symptoms. Previous reports indicate that it might require weeks to months for the condition to resolve [14]. Treatment in this pregnant patient with a corticosteroid taper resulted in a rapid and effective response, with no apparent impact on the term fetus, after other conservative measures failed.

## Consent

Written informed consent was obtained from the patient for the publication of this case report. A copy of the written consent is available for review by the Editor-in-Chief of this journal.

## Abbreviations

CSF: Cerebrospinal fluid; CT scan: Computed tomography scan; G2P1: Gravida 2 para 1; IV: Intravenous; MRI: Magnetic resonance imaging; SIH: Spontaneous intracranial hypotension.

## Competing interests

The author declares that she has no competing interests.
